# NAD^+^ Therapeutics and Skeletal Muscle Adaptation to Exercise in Humans

**DOI:** 10.1007/s40279-022-01772-2

**Published:** 2022-11-04

**Authors:** Dean Campelj, Andrew Philp

**Affiliations:** 1 Biology of Ageing Laboratory, Centenary Institute, Missenden Road, Camperdown, Sydney, NSW 2050 Australia; 2 Centre for Healthy Ageing, Centenary Institute, Missenden Road, Sydney, NSW Australia; 3 Faculty of Medicine and Health, Charles Perkins Centre, University of Sydney, Sydney, NSW Australia; 4 Faculty of Health, School of Sport, Exercise and Rehabilitation Sciences, University of Technology Sydney, Ultimo, NSW Australia

## Abstract

Nicotinamide adenine dinucleotide (NAD^+^) is a vital energy intermediate in skeletal muscle. The discovery of dietary-derived NAD^+^ precursors has led to the rapid development of NAD^+^ therapeutics designed to manipulate NAD^+^ content in target tissues. Of those developed, nicotinamide riboside and nicotinamide mononucleotide have been reported to display health benefit in humans under clinical scenarios of NAD^+^ deficiency. In contrast, relatively little is known regarding the potential benefit of nicotinamide riboside and nicotinamide mononucleotide supplementation in healthy individuals, with questions remaining as to whether NAD^+^ therapeutics can be used to support training adaptation or improve performance in athletic populations. Examining animal and human nicotinamide riboside supplementation studies, this review discusses current evidence suggesting that NAD^+^ therapeutics do not alter skeletal muscle metabolism or improve athletic performance in healthy humans. Further, we will highlight potential reasons why nicotinamide riboside supplementation studies do not translate to healthy populations and discuss the futility of testing NAD^+^ therapeutics outside of the clinical populations where NAD^+^ deficiency is present.

## Key Points

**Table:** 

Nicotinamide adenine dinucleotide is a key energy intermediate in skeletal muscle thought to promote mitochondrial adaptation to endurance exercise.
Cellular nicotinamide adenine dinucleotide levels can be altered by the dietary-derived precursors tryptophan, nicotinic acid, nicotinamide, nicotinamide riboside, and nicotinamide mononucleotide.
Supplementation of nicotinamide riboside increases mitochondrial biogenesis and functional performance in rodent models of nicotinamide adenine dinucleotide deficiency such as aging, obesity, and mitochondrial myopathy.
Nicotinamide riboside alters the NAD^+^ metabolome in human skeletal muscle but does not increase mitochondrial biogenesis or augment intracellular signaling processes following a single bout of endurance exercise or prolonged endurance training.
Current evidence suggests that NAD^+^-targeted therapeutics do not improve training adaptation or athletic performance in humans.
Future work should establish physiological conditions where NAD^+^ deficiency occurs to identify potential scenarios where NAD^+^-targeted therapeutics may have a benefit in humans.

## Introduction

Nicotinamide adenine dinucleotide (NAD^+^) is a ubiquitous cellular coenzyme, first discovered in 1906 [1]. NAD^+^ is a key metabolic intermediate, capable of harnessing energy from glucose, fatty acids, and amino acids via glycolysis, β-oxidation, and the tricarboxylic acid cycle [2]. In recent years, a role for NAD^+^ as a signaling moiety that can impact numerous biological processes has emerged, primarily owing to the discovery of cellular NAD^+^ sensors that can mediate metabolic remodeling in skeletal muscle in response to alterations in NAD^+^ availability [2]. Accordingly, this area of biology has become a hot topic of investment for biotech companies seeking to explore NAD^+^ boosters as potential dietary supplements for a variety of metabolic diseases characterized by NAD^+^ deficiency. The aim of this review is to (i) provide an overview of NAD^+^ metabolism in skeletal muscle, (ii) discuss how diet and exercise interact to alter NAD^+^ content, (iii) highlight different oral supplementation approaches to manipulate NAD^+^ in vivo, and (iv) examine the feasibility of NAD^+^-targeted therapeutics to improve athletic performance in humans.

## The Role of NAD^+^ in Skeletal Muscle

The term metabolism describes the sum of all the reactions within the body that transduce chemical energy for cellular processes from ingested substrate stores [3, 4]. The most prevalent of these reactions in skeletal muscle is the catabolism of dietary lipid, carbohydrate, or protein stores to generate adenosine triphosphate (ATP), the energy unit of the cell. During endurance exercise, the majority of energy is derived from lipid and carbohydrate stores. This process is tightly regulated, with numerous feedback and feed-forward mechanisms ensuring that the cell can switch between lipid and carbohydrate metabolism when required. The key determinant in the selection of carbohydrate or lipid metabolism as the preferential fuel source is the intensity (how hard) and the duration (how long) of muscle contraction. As a 6-carbon molecule, glucose, the principal dietary carbohydrate, is readily metabolized without the requirement of oxygen, to pyruvate during a ten-step enzyme process termed glycolysis [3, 4]. The formation of pyruvate is key to the progression of metabolism as two reactions stem from this metabolite, first, the generation of lactate, via lactate dehydrogenase, and second, the production of acetyl-CoA via pyruvate carboxylation via the pyruvate dehydrogenase complex. The latter is particularly important as acetyl-CoA, following conversion to citrate forms the principal donor for the tricarboxylic acid cycle, which initiates the cycling of carbon substrate to generate electrons (NAD^+^ and flavin adenine dinucleotide) for the final stages of oxidative phosphorylation and the synthesis of ATP [3, 4].

During the transition from rest to exercise, the metabolic requirement of mammalian skeletal muscle for ATP synthesis can increase over 100-fold, while the ATP level only decreases marginally [5]. To maintain ATP homeostasis, the pathways of ATP synthesis must be activated rapidly to match the rate of ATP utilization [5]. Glycolysis and oxidative phosphorylation provide most of the ATP used during exercise; in both processes, the interconversion between NAD^+^ and, its reduced equivalent, NADH, occurs [6]. This redox couple not only participates in reduction–oxidation reactions as co-substrates, but also plays important roles in metabolic regulation in both the cytosol and mitochondria [2, 5]. NADH produced in glycolysis is utilized in reactions catalyzed by lactate dehydrogenase, or transported to the mitochondria for oxidation in the electron transport chain to maintain the redox states (NADH/NAD^+^) in cytosol and mitochondria [2, 5]. With significantly increased metabolic rates during exercise, it is therefore crucial to maintain the balance of NADH and NAD^+^ within cytosolic and mitochondrial compartments [2, 5].

## Mechanisms of NAD^+^ Turnover and Homeostasis

**Fig. 1:**
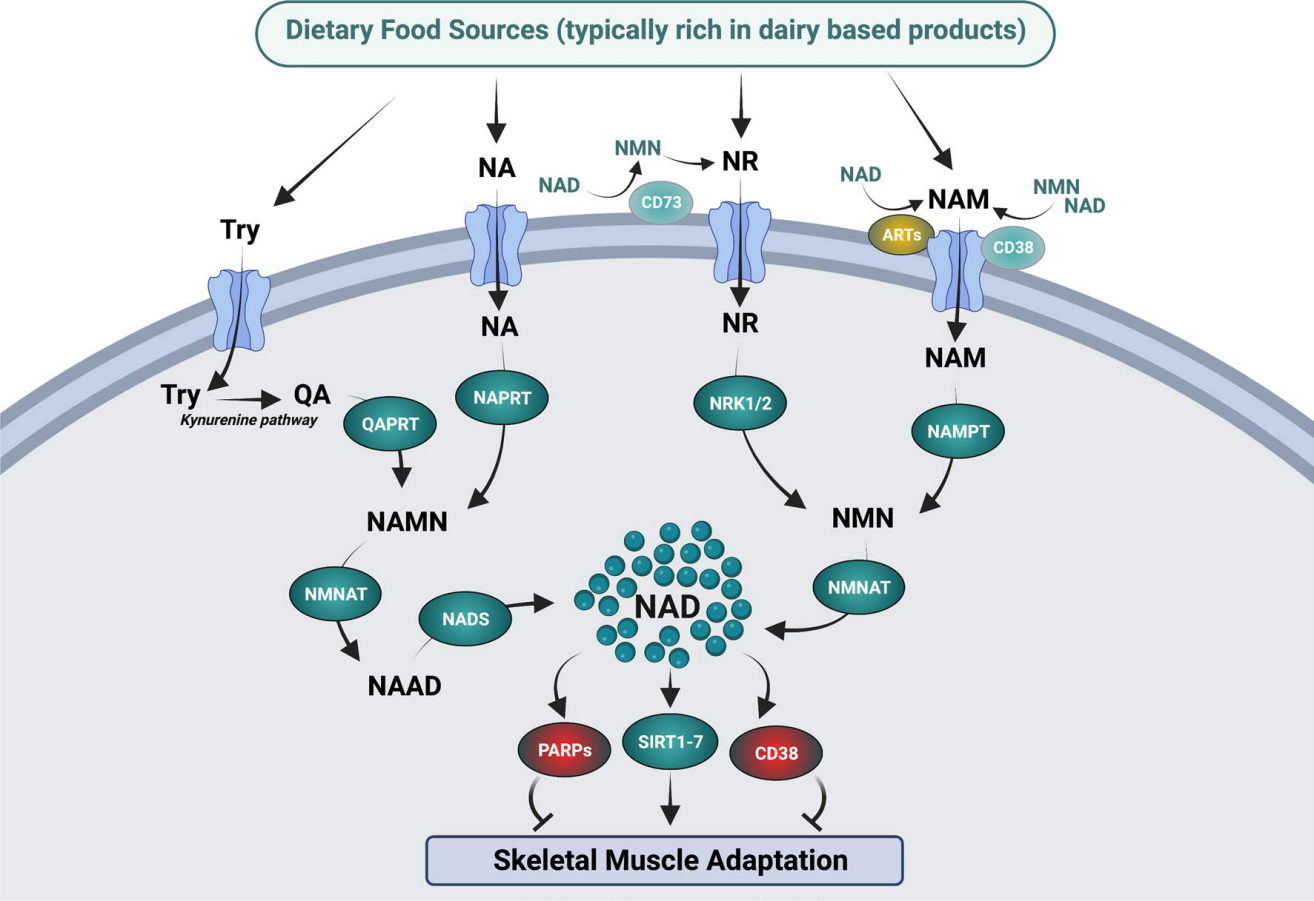
Influence of dietary-derived nicotinamide adenine dinucleotide (NAD^+^) precursors on the NAD^+^ metabolome in skeletal muscle. The supplementation of the NAD^+^ donors tryptophan (Try), nicotinic acid (NA), nicotinamide riboside (NR), and nicotinamide (NAM) activates a regulatory cascade of homeostatic mechanisms that converge to alter cellular levels of NAD^+^. The de novo synthesis pathway involves the metabolic break down of Try and NA metabolic pathways. Try is converted into quilonic acid via the kynurenine pathway, before undergoing phosphoribosylation from quinolinic acid phosphoribosyltransferase (QAPRT), to produce nicotinic acid mononucleotide (NAMN). Nicotinic acid is also converted into NAMN, as it undergoes phosphoribosylation from NA phosphoribosyltransferase (NAPRT). Following these reactions, NAMN is converted into nicotinic acid adenine dinucleotide (NAAD), via nicotinamide mononucleotide adenylyltransferase (NMNAT) activity. NAAD then undergoes amidation via NAD^+^ synthetase (NADS) to drive NAD^+^ synthesis. The NAD^+^ salvage pathway involves the metabolic break down of NR and NAM. NR and NAM can be sourced through the diet or via the activation of the NAD^+^ and nicotinamide mononucleotide (NMN) degrading ectoenzymes; CD73 and CD38, respectively. Free NAM can also be sourced via activation of ADP-ribosyltransferases (ARTs). Once NR and NAM cross the plasma membrane, they are converted into NMN. NR undergoes phosphorylation by the NR kinases 1 and 2 (NRK1 and NRK2), while NAM is modified through phosphoribosylation by NAM phosphoribosyltransferase (NAMPT). NMN is then converted into NAD^+^ via NMNAT. The activation or suppression of these metabolic cascades can influence NAD^+^ levels, which in turn alters the activity of the NAD^+^ sensors sirtuins (SIRTs) 1–7, to promote skeletal muscle adaptation. In parallel, the NAD^+^ consuming proteins PARP1/2 and CD38 compete with SIRTs for NAD^+^ and are thought to suppress skeletal muscle adaptation. Image created by BioRender.com

As with any metabolic intermediate, NAD^+^ content in skeletal muscle is dictated by processes of production and consumption, which maintain the NAD^+^ pool. The main processes of NAD^+^ generation in skeletal muscle can be separated into two classes, (i) de novo synthesis and (ii) salvaging pathways. Importantly, NAD^+^ is sensitive to dietary manipulation, as tryptophan (Try), nicotinic acid (NA), nicotinamide riboside (NR), and nicotinamide (NAM) can all influence NAD^+^ content (Fig. 1). The de novo synthesis pathway is prevalent in mammalian systems, and functions to convert the amino acid Try into quinolinic acid and then subsequently into nicotinic acid mononucleotide (NAMN). The conversion to NAMN is important as this then serves as a precursor for NAD^+^ (Fig. 1). Despite Try being the canonical NAD^+^ precursor, its action is thought to be up to 60 times less efficient than NA, as Try is also used for protein translation and biosynthetic processes [7]. The recommended daily allowance of NAMN derived from NA is 16 mg/day for men and 14 mg/day for women, suggesting that targeting of NAMN from NA is more effective than the substantial amount of Try that would be required (Try 960 mg/day for men) [8]. As such, the use of Try as a NAD^+^ precursor would not be solely sufficient to support physiological NAD^+^ requirements in mammals [9].

In contrast to Try, NA is an established dietary approach to synthesize NAD^+^ (Fig. 1). Once inside the cell, NA is converted to NAD^+^ through the Preiss–Handler pathway, in a three-step process involving the phosphoribosylation of NA to NAMN, NAMN adenylation to nicotinic acid adenine dinucleotide, and nicotinic acid adenine dinucleotide amidation to NAD^+^ by the glutamine-dependent NAD^+^ synthetase [10]. Whilst NA supplementation was initially viewed as a feasible approach to boost NAD^+^ levels in vivo, the clinical use of NA has been hindered by observations that NA ingestion results in a pronounced flushing response. This flushing effect appears to be mediated by the binding of NA to the G protein-coupled receptor, GPR109A (HM74A or PUMA-G) [11]. However, in contrast to Try, NA is a potent NAD^+^ precursor in the liver and kidneys, primarily because nicotinate phosphoribosyltransferase enzymatic activity is exaggerated in these tissues [12]. There is also evidence in vivo that the majority of dietary-derived NA is rapidly metabolized into NAM in the gut and the liver [13], which consequently results in a low circulatory abundance of NAM in plasma [12]. Collectively, these data suggest that Try and NA supplementation would have a limited effect on boosting NAD^+^ in vivo when provided as a dietary supplement, owing to inefficient synthesis (Try) or excessive clearance via the liver (NA).

The final routes of NAD^+^ synthesis in mammals are provided through the NAD^+^ recycling/salvage pathways supported by the NAD^+^ precursors NAM and NR. NAM is converted into NAD^+^ initially via the phosphoribosylation action of the enzyme NAM phosphoribosyltransferase (NAMPT), which converts NAM to the intermediate NMN. NMN then undergoes conversion to NAD^+^, mediated by NMN acetyltransferase (Fig. 1) [10]. NAM appears to have a high turnover rate, given it is a product of NAD^+^ degradation in a constant cycle of breakdown and recycling, and thus, unsurprisingly, it has a higher concentration in skeletal muscle compared with NAD^+^ [14]. This suggests that NAMPT is an integral component of NAD^+^ metabolism. For example, transgenic overexpression of NAMPT leads to an increase in skeletal muscle NAD^+^ levels [15–17], whilst muscle-specific deletion of NAMPT leads to NAD^+^ depletion and a muscle dystrophic phenotype [18, 19]. In contrast, NR can be transported into the cell by nucleoside transporters [20], before undergoing phosphorylation by the NR kinases 1 and 2 (NRK1 and NRK2) to generate NMN [20]. Once formed, NMN is then converted to NAD^+^ via NMN acetyltransferase.

## How is NAD^+^ Metabolism Altered Under Different Physiological Conditions?

NAD^+^ is thought to have distinct cytosolic, mitochondrial, and nuclear pools within skeletal muscle [21]. However, typically, NAD^+^ content is reported as whole cell values, offering little distinction between the regional sites of NAD^+^ abundance. Further, few studies have examined NAD^+^ turnover in cell systems using stable-isotope tracer approaches, meaning that very little is known about dynamic movement of NAD^+^ and associated NAD^+^ metabolites within the cell [21]. Consequently, very little is known about NAD^+^ cellular levels or localization in skeletal muscle [2].

In resting human muscle, total NAD^+^ and NADH^+^ concentrations are estimated to be ~ 1.5–1.9 and ~ 0.08–0.20 mmol/kg dry weight muscle, respectively [2], 10% of which would be assumed to be mitochondrial [22]. Accordingly, the NAD^+^/NADH ratio in resting skeletal muscle is estimated to be greater in the cytosol compared with mitochondria [2]. During maximal exercise, i.e., above 100% of maximal oxygen uptake (*V*O_2_max), NADH has been reported to increase approximately two-fold above resting levels, with no significant change reported in NAD^+^ levels [23, 24]. In contrast, during submaximal exercise, total muscle NADH concentrations are not thought to change during exercise at 75% *V*O_2_max [25] or decrease during exercise at 50% *V*O_2_max [26]. Exercise intensity appears to be an important contributor to the differences in measured NAD^+^(H) and NAD^+^/NADH ratio during exercise. For example, NADH decreased (and the cytosolic NAD^+^/NADH ratio was unchanged) from resting values during exercise at 40% *V*O_2_max, but both NAD^+^ and the cytosolic NAD^+^/NADH ratio were increased above resting values at 75% and 100% *V*O_2_max [27].

## Do Cellular NAD^+^ Sensors Drive Skeletal Muscle Mitochondrial Adaptation to Exercise?

Interest in NAD^+^ donors as skeletal muscle therapeutics has stemmed primarily because of the identification and characterization of sirtuin 1 (SIRT1), a cellular NAD^+^-activated metabolic sensor and purported regulator of skeletal muscle oxidative capacity. Over the past decade, SIRT1 has been shown to influence the activity of the AMP-activated protein kinase and the transcriptional co-activator, peroxisome proliferator activated receptor-gamma coactivator-1α (PGC-1α) in vitro, representing a metabolic sensing node, translating fluctuations in cellular stresses to mitochondrial remodeling [28].

The first mechanistic link between SIRT1 and mitochondrial adaptation was provided by Nemoto and colleagues, who demonstrated that SIRT1 deacetylates and activates PGC-1α in an NAD^+^-dependent manner in PC12 neuron-like cells [29]. In parallel, Rodgers and colleagues reported that SIRT1 and PGC-1α protein content were rapidly up-regulated in the liver following fasting and returned to basal conditions upon re-feeding [30], mirroring increased hepatic pyruvate and NAD^+^ concentrations. Following the observation that PGC-1α was deacetylated following fasting, the authors proposed that fasting increased cellular NAD^+^ concentrations that initiated an increase in the SIRT1 and PGC-1α association to increase gluconeogenic gene expression [30]. With regard to skeletal muscle, Gerhart-Hines et al. reported that overexpression of SIRT1 led to increased expression of genes involved in mitochondrial respiration and fatty acid utilization in C2C12 myotubes [31]. Further, short hairpin RNA knockdown of SIRT1 reduced the expression of cytochrome-c, isocitrate dehydrogenase-3α, cytochrome-c oxidase subunit IV, medium-chain acyl-CoA dehydrogenase, carnitine palmitoyltransferase-1, pyruvate dehydrogenase kinase-4, and citrate synthase activity, in both C2C12 and mouse primary myotubes [31]. Further, the authors demonstrated that increased mitochondrial metabolic activity in vitro in response to glucose reduction was SIRT1 dependent, as both SIRT1 knockout mouse embryonic fibroblasts and SIRT1 short hairpin RNA knockdown blocked the increase in mitochondrial gene expression and fatty acid utilization observed in wild-type cells [31]. Collectively, these studies established the paradigm that SIRT1 was a key signaling node in vitro, linking alterations in cellular energy status to increased mitochondrial adaptation and β-oxidation, through the targeted deacetylation and activation of PGC-1α.

In contrast to the in vitro studies described above, a role for SIRT1 in the regulation of skeletal muscle mitochondrial biogenesis in vivo is not as clear. For example, Gurd and colleagues reported that a single bout of exhaustive exercise and 7 days of electrical stimulation reduced whole-muscle or nuclear SIRT1 protein content, yet increased whole-muscle and nuclear PGC-1α protein content and SIRT1 activity, respectively [32, 33]. The clearest disconnect between SIRT1 activity and mitochondrial adaptation to endurance exercise has been provided using SIRT1 muscle-specific knockout mice [34], demonstrating that mitochondrial adaptation to acute and chronic exercise is preserved in the absence of SIRT1 activity. Further, muscle-specific SIRT1 overexpression leads to incomplete mitochondrial biogenesis [35] and even a reduction in cytochrome-c oxidase subunit IV and PGC-1α protein content and SIRT1 activity [32]. Thus, when considering these findings together, it appears that SIRT1 is not a critical regulator of skeletal muscle mitochondrial adaptation to exercise, whilst super-physiological increases in SIRT1 can impair mitochondrial function in skeletal muscle.

## Nutraceutical Approaches to Target NAD^+^ Metabolism In Vivo

Nutraceuticals that target NAD^+^ metabolism have materialized with great interest to the scientific community, primarily involving synthetic precursors of NAD^+^, i.e., vitamin B_3_ [36]. Niacin is one of the recognized forms of vitamin B_3_, a compound constituting both NA and NAM [37, 38]. Supplementation of niacin (i.e., acipimox) resulted in modest but significant increases in state 3 and maximal respiration, increased ATP content, and increased the protein content of mitochondrial electron transport chain complexes 1 and 2 and heat-shock protein 60 [39]. Unfortunately, the authors did not assess NAD^+^ metabolism or skeletal muscle oxidative capacity, making it difficult to draw conclusions on the direct or indirect mechanism responsible for the mitochondrial adaptation observed from acipimox supplementation.

Regarding NMN action in vivo, a recent clinical trial examined the effect of supplementing 250 mg/day over 10 weeks in pre-diabetic women. Of note, this period of supplementation led to modulation of the skeletal muscle NAD^+^ metabolome and significant improvements in insulin sensitivity [40]. Less is currently known regarding NMN supplementation and adaptation to exercise. Mills et al. have reported that NMN supplementation at either 100 or 300 mg/kg/day over 12 months increased physical activity in otherwise healthy mice [41], whilst 500 mg/kg/day for 1 month improved exercise capacity in aged mice [42]. Further, a recent human trial of NMN supplementation at 300, 600, and 1200 mg/kg/day over 6 weeks, alongside endurance exercise training, displayed modest effects on whole-body aerobic adaptations. Specifically, there was no change observed to *V*O_2max_ but an increase in power and oxygen uptake at the ventilatory threshold [43].

## NR and Skeletal Muscle Adaptation to Exercise

NR is a naturally occurring form of vitamin B_3_, a single chemical moiety containing NAM and ribose that is found in milk, meat, poultry, fish, eggs, and green vegetables [44]. As an NAD^+^ precursor, NR is thought to impact skeletal muscle mitochondrial function through stimulation of the NAD^+^/SIRT1/PGC-1α signaling cascade [7]. The first evidence for the direct effect of NR on skeletal muscle metabolic function was provided by Canto and colleagues who showed that NR supplementation to C2C12 myotubes increases NAD^+^ content in the absence of GPR109A mobilization [45]. Feeding NR to mice (400 mg/kg/day) resulted in modest increases in skeletal muscle NAD^+^ (~ 5%) following 1-week supplementation, in a mechanism independent of changes in NAM phosphoribosyltransferase expression [45]. The authors proposed that the metabolic action of NR supplementation was mediated through SIRT1, as the adaptive response of C2C12 myotubes to NR supplementation was lost when SIRT1 was deleted. Interestingly, NR supplementation protected mice from the deleterious effects of 8 weeks of high-fat feeding, principally through an increase in energy expenditure and a reduction in cholesterol levels [45]. However, the ability of NR to protect against a high-fat diet has recently been challenged by two independent groups [46, 47], who were unable to replicate the findings of Canto et al. [45]. In parallel with metabolic adaptation, Canto et al. reported that endurance capacity also increased by ~ 25% in the NR supplemented mice, coupled with an increase in mitochondrial copy number and increased mitochondrial electron transport chain protein content, concluding that NR supplementation could promote modest increases in skeletal muscle NAD^+^ content and mitochondrial biogenesis [45]. Recently, Damgaard and colleagues provided contrasting data to Canto et al., reporting that NR supplementation had no impact on mitochondrial respiratory capacity or mitochondrial gene transcription following exercise training in mice, despite increased skeletal muscle NAD^+^ content [48]. Thus, current evidence suggests that NR supplementation does not promote mitochondrial adaptation to exercise in lean or obese mice. Subsequent research has demonstrated that NR supplementation can improve skeletal muscle NAD^+^ content and mitochondrial function in mouse models of mitochondrial myopathy [49, 50] and Duchenne muscular dystrophy [51], although again, the validity of this study has recently been challenged [52].

**Fig. 2:**
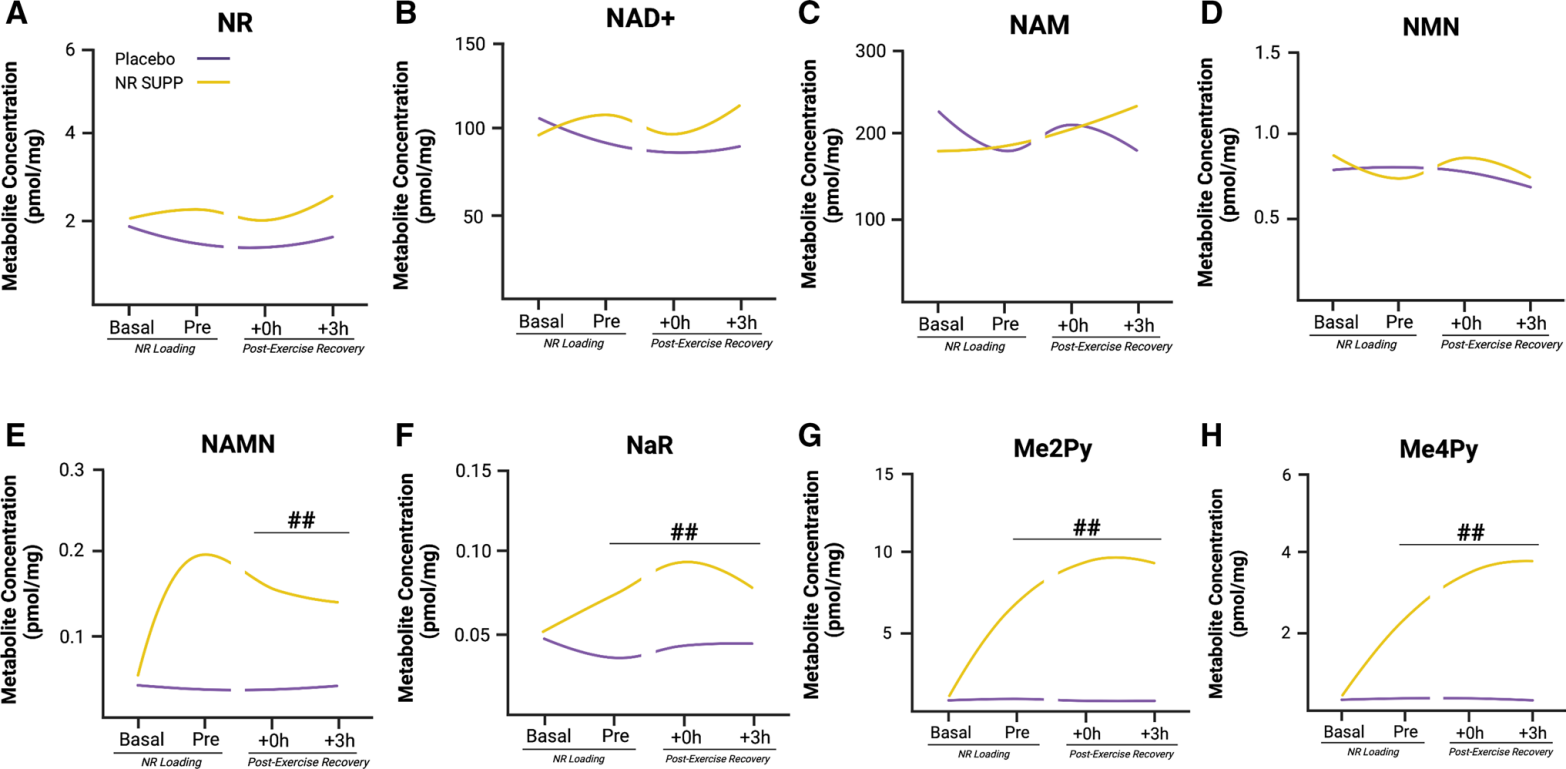
Effect of nicotinamide riboside (NR) loading and acute endurance exercise on the skeletal muscle nicotinamide adenine dinucleotide (NAD^+^) metabolome. The figure shows the effects of seven days of NR loading (NR SUPP) versus cellulose placebo on NAD^+^ metabolism in young male volunteers prior to an acute bout of endurance exercise. Specifically, NR loading had no effect on NR, NAD^+^, nicotinamide (NAM), and nicotinamide mononucleotide (NMN) content in skeletal muscle (**A**–**D**). However, deaminated NAD^+^ precursor, nicotinic acid riboside (NaR), and, methylated breakdown products, N-methyl-2-pyridone-5-carboxamide (Me2Py) and N-methyl-4-pyridone-5-carboxamide (Me4Py), were elevated following NR loading and sustained during the 3-h post-exercise period (**E**–**G**; ## denotes *p* < 0.05 placebo vs NR SUPP). Interestingly, the phosphoribosylated NAD^+^ precursor, nicotinic acid mononucleotide (NAMN), was only increased post-NR supplementation and exercise (**H**), whilst exercise alone had no effect on the NAD + metabolome in skeletal muscle (**A**–**G**). *h* hours. Image created by BioRender.com

Despite the promise observed in pre-clinical models, the NAD^+^ boosting effects of NR supplementation have been associated with limited translational progress in humans to date. The most conclusive examination of NR supplementation and skeletal muscle adaptation to training has been provided by Dollerup and colleagues, who demonstrated that NR supplementation (1 g twice daily for 12 weeks) in obese humans with insulin resistance was unable to alter NAD^+^ and its reduced equivalents in skeletal muscle and had no effect on mitochondrial protein abundance and respiration [53]. These observations align with the work from Elhassan et al., who reported that NR supplementation (1 g daily for 3 weeks) in older male individuals (median age of 75 years) did not increase mitochondrial respiration or citrate synthase activity in skeletal muscle [54]. Interestingly, this study did however demonstrate that NR supplementation could alter the NAD^+^ metabolome in skeletal muscle, with a liquid chromatography-mass spectrometry analysis reporting an increase in the NAMN derivative nicotinic acid adenine dinucleotide, and the methylated NAM break-down products methylated NAM and N-methyl-2-pyridone-5-carboxamide (Me2PY) [54]. Unfortunately, the absence of a young cohort in this study meant that it was not possible to compare the effect of age on the skeletal muscle NAD^+^ metabolome, or determine a physiological context to the relevance of the changes in the NAD^+^ metabolome. Recently, we have investigated the effect of short-term NR loading (1 g daily supplementation for 1 week) on the NAD^+^ metabolome and related intracellular signaling prior to and following a single bout of exercise in young healthy male volunteers [55]. Consistent with our previous work in older adults [54], NR loading did not alter the metabolite concentrations of NAD^+^, NR, NAM, and NMN in skeletal muscle (Fig. 2A–D); however, NR loading did increase the concentration of the NAD^+^ precursor, nicotinic acid riboside, and, methylated NAM break-down products, Me2PY and N-methyl-4-pyridone-5-carboxamide (Me4PY), which was sustained immediately and 3 h post-exercise (Fig. 2E–H). Interestingly, we observed that the NAD^+^ precursor NAMN was elevated immediately and 3-h post-exercise. Whilst NR loading was able to augment some aspects of the skeletal muscle NAD^+^ metabolome, we observed no additive effect to exercise in relation to substrate utilization during and in recovery from exercise, nor did we see any amplification in SIRT1 or SIRT3 signaling, or the messenger RNA expression of PGC-1α in the post-exercise recovery period [55].

## Why Does NR Not Augment Mitochondrial Adaptation in Human Skeletal Muscle?

Based on the data discussed above, two key questions emerge, (i) why the disparity between cell, animal, and human studies? and (ii), why does NR not alter skeletal muscle metabolism, training adaptation, or athletic performance in humans? Regarding the disparity between rodent and human studies, it appears that NAD^+^ precursors such as NR are most effective when supplemented in a scenario of NAD^+^ deficiency (i.e., mitochondrial myopathy). In this context, NR can reverse this NAD^+^ deficiency and improve metabolic health. However, we are not aware of any studies that have shown a benefit of NR supplementation in young healthy animal models, thus it is not surprising that NR does not have a huge effect in healthy human volunteers in basal conditions, given that there is no compromise in NAD^+^ metabolism. Similarly, the rapid turnover and replenishment of NAD^+^ during exercise would also lead to the assumption that NR supplementation would have marginal effects if any during exercise, a statement supported by our recent work [55]. Differences in the impact of NR supplementation between animals and human studies may also be influenced by the scale of dosing between species [56] and the dosing strategy [14], i.e., dose matching, route of administration, and timing [48]. Overall, current evidence would suggest that NR supplementation can only be beneficial in scenarios of rescuing NAD^+^ deficiency.

The question as to why NR supplementation does not augment training adaptation or athletic performance in humans is also relevant for discussion. The mechanism thought to link NR and skeletal muscle mitochondrial biogenesis is through the SIRT1/PGC-1α signaling cascade, which is proposed to be activated by increased NAD^+^ availability during and in recovery from exercise. However, there is limited evidence for this pathway to directly mediate skeletal muscle adaptation to endurance exercise in both rodents and humans [28]. For example, we have previously shown that exercise-induced mitochondrial adaptation to endurance exercise training is preserved in muscle-specific SIRT1 knockout mice [34], whilst muscle-specific SIRT1 overexpression in skeletal muscle leads to incomplete mitochondrial biogenesis [35, 57]. In addition, mitochondrial adaptation to endurance exercise training is preserved in skeletal muscle-specific PGC-1α knockout mice [58]. There are also very limited data to support that endurance exercise promotes SIRT1 activation in human skeletal muscle [28, 59]. We recently reported that neither exercise nor exercise in combination with NR loading increased SIRT1 or SIRT3 signaling in human skeletal muscle, despite clear activation of PGC-1α gene expression in the 3-h period post-exercise [55]. Therefore, despite general acceptance for SIRT1 to be an important player in skeletal muscle adaptation to endurance exercise, direct support for this role is lacking and could explain why NR loading is largely ineffective at augmenting adaptation to training as SIRT1 and SIRT3 display substantial redundancy during skeletal muscle adaptation to exercise.

## Conclusions and Future Directions

NAD^+^ therapeutics represent a novel nutritional approach to potentially manipulate skeletal muscle mitochondrial function and metabolic adaptation to endurance exercise. However, despite promising data in cell and rodent studies, the potential of various NAD^+^ therapeutics has yet to translate in humans unless clear NAD^+^ deficiency is present [50]. NAD^+^ metabolism in skeletal muscle, unlike other tissues, appears to be tightly regulated, with limited evidence suggesting that NAD^+^ deficiency occurs in physiological scenarios of relevance. Thus, based on the present literature, there does not appear to be a scenario currently identified where NR supplementation could be used to benefit skeletal muscle adaptation or athletic performance in vivo. Therefore, if researchers and practitioners are to further explore avenues where NAD^+^ therapeutics might be of benefit, it seems prudent that the focus should first be placed on determining physiological scenarios where NAD^+^ deficiency is apparent before examining the ergogenic benefit of NAD^+^-targeted therapeutics.
